# Understanding Mental Health Professionals’ Perspectives and Practices Regarding the Implementation of Digital Mental Health: Qualitative Study

**DOI:** 10.2196/32558

**Published:** 2022-04-12

**Authors:** Cristina Mendes-Santos, Francisco Nunes, Elisabete Weiderpass, Rui Santana, Gerhard Andersson

**Affiliations:** 1 Department of Culture and Society Linköping University Linköping Sweden; 2 NOVA National School of Public Health Public Health Research Centre Universidade Nova de Lisboa Lisbon Portugal; 3 Fraunhofer Portugal AICOS Porto Portugal; 4 Experimental Pathology and Therapeutics Group Portuguese Institute of Oncology Porto Portugal; 5 International Agency for Research on Cancer Lyon France; 6 Department of Behavioural Sciences and Learning Linköping University Linköping Sweden; 7 Department of Clinical Neuroscience, Psychiatry Section Karolinska Institutet Stockholm Sweden

**Keywords:** barriers, digital mental health, drivers, implementation, internet interventions, psychotherapy, technology acceptance and adoption, mental health professionals, Portugal, European Union, EU

## Abstract

**Background:**

Despite the potential of digital mental health to provide cost-effective mental health care, its adoption in clinical settings is limited, and little is known about the perspectives and practices of mental health professionals regarding its implementation or the factors influencing these perspectives and practices.

**Objective:**

This study aims to characterize in depth the perspectives and practices of mental health professionals regarding the implementation of digital mental health and explore the factors affecting such perspectives and practices.

**Methods:**

A qualitative study using in-depth semistructured interviews with Portuguese mental health professionals (N=13)—psychologists and psychiatrists—was conducted. The transcribed interviews were thematically analyzed.

**Results:**

Mental health professionals deemed important or engaged in the following practices during the implementation of digital mental health: indication evaluation, therapeutic contract negotiation, digital psychological assessment, technology setup and management, and intervention delivery and follow-up. Low-threshold accessibility and professionals’ perceived duty to provide support to their clients facilitated the implementation of digital mental health. Conversely, the lack of structured intervention frameworks; the unavailability of usable, validated, and affordable technology; and the absence of structured training programs inhibited digital mental health implementation by mental health professionals.

**Conclusions:**

The publication of practice frameworks, development of evidence-based technology, and delivery of structured training seem key to expediting implementation and encouraging the sustained adoption of digital mental health by mental health professionals.

## Introduction

### Background

Digital mental health may be understood as the use of digital technologies to support and improve mental health conditions and provide mental health care, including screening, health promotion, prevention, early intervention, treatment, and relapse prevention [1-3]. It encompasses a wide range of modalities, including internet research [4], monitoring and assessment [5], videoconferencing counseling and psychotherapy [5], internet interventions [6], and professional training (e-learning and e-supervision) [7]. In this regard, various technologies (telephone, mobile devices, apps, videoconference and chat software, psychological assessment, support and intervention platforms, artificial intelligence, virtual reality, serious games, wearable devices, etc) may be used to improve health outcomes or facilitate health care service delivery [1,2].

Owing to the potential of digital mental health to increase access to mental health care, eliminate disparities, and reduce costs of treatment delivery, interest and research in the field have grown exponentially in the past years [8]. Numerous randomized controlled trials have been conducted, and a strong body of evidence of efficacy has been generated [9,10], particularly in the domains of internet interventions [11] and videoconferencing counseling and psychotherapy [12].

Internet interventions are self-help, guided or unguided, technology-enabled interventions that aim to provide health and mental health–related assistance [11]. They have been found to be more effective than treatment as usual and as effective as face-to-face therapies for various conditions [13] (eg, generalized anxiety disorder [14], depression [15], and cancer-related distress [16]). The impact of guidance on the efficacy of internet interventions has also been examined. Although previous studies suggest that guided interventions are superior to unguided interventions [17], there is still a lack of research comparing the outcomes of blended treatment outcomes with classic face-to-face or nonblended treatments [18]. Internet interventions have also been found to be cost-effective when compared with various control conditions, such as active, attention, or waiting list control groups [19].

With regard to counseling and psychotherapy delivered via videoconference, previous systematic reviews [12,20] have reported that they can be effective across different populations (eg, children, adults, and older adults), geographies (eg, urban and rural), care settings (eg, primary health care settings and clinics), and mental health conditions (eg, anxiety, depression, and distress). Previous studies have reported that the effects are comparable with both in-person treatment and blended care approaches [20], and evidence on videoconferencing counseling and psychotherapy cost-effectiveness is increasing [21]. However, although sound scientific support favors the use of digital mental health approaches for the treatment of mental health disorders, its adoption by mental health professionals has been slow, and its implementation in clinical settings is still limited [22]. In Portugal, despite a significant mental health treatment gap [23,24], digital mental health initiatives are practically nonexistent [25].

Across the globe, various studies have been conducted to identify potential drivers of and barriers to digital mental health adoption (ie, acceptance, uptake, and use) and investigate mental health professionals’ attitudes toward such an approach [26]. In general, findings suggest that professionals’ attitudes range from neutral to generally positive [27], and there are several factors affecting adoption. Factors that are often identified as expediting adoption relate to the low-threshold accessibility of digital mental health [28,29], professionals’ knowledge and training in the field [30,31], the potential to introduce new treatment alternatives (eg, virtual reality and biofeedback) [32], and professionals’ positive attitudes toward digital mental health [28]. Conversely, factors frequently appointed as inhibiting adoption relate to the absence of ethical, legal, and regulatory frameworks for providing web-based mental health care [33-36]; professionals’ lack of knowledge and training in the field [28,30,31]; potential confidentiality and security breaches associated with digital systems [37]; and negative attitudes toward digital mental health [28]. Furthermore, greater acceptance of blended care approaches was reported across studies [22,32].

Although previous research provides valuable insight into the factors influencing the adoption of digital mental health by professionals, adoption predictors, and their interrelationships are largely unknown. Most studies with health care professionals adopt a quantitative cross-sectional design, capturing the stance of large samples and listing implementation drivers and barriers but failing to provide an in-depth understanding of therapists’ experiences, attitudes, and adoption determinants [32]. Moreover, qualitative studies on the topic often focus on cognitive behavioral therapy–oriented interventions [38,39], specific treatment modalities [39,40], and particular mental health conditions [22,29,39] or include participants of specific research programs [22,29,39,41], failing to characterize in depth the perspectives and practices of mental health professionals regarding the implementation of digital mental health or the factors influencing such perspectives and practices.

### Objective

The aims of this study are (1) to characterize in depth the perspectives of mental health professionals regarding digital mental health, (2) to characterize in depth the practices of mental health professionals regarding the implementation of digital mental health, and (3) to explore the factors influencing such perspectives and practices in the context of Portugal.

## Methods

### Study Design

This qualitative study used in-depth semistructured interviews to characterize the perspectives and practices of Portuguese mental health professionals regarding the implementation of digital mental health and explore the factors influencing such perspectives and practices. A semistructured interview guide (Multimedia Appendix 1) was developed based on a literature review and analysis of data obtained from a previous study by the research team [28]. The interview guide included 33 questions and covered five main domains: (1) professional background and digital technology proficiency, (2) knowledge and use of digital mental health, (3) attitudes toward digital mental health, (4) advantages and limitations of digital mental health, (5) drivers of and barriers to the adoption and implementation of digital mental health, and (6) therapeutic process and alliance in digital mental health interventions. In addition, follow-up questions were used to clarify participants’ perspectives and practices. The COREQ (Consolidated Criteria for Reporting Qualitative Research) checklist was used as a guideline to structure this paper [42].

### Sampling and Recruitment

A nonprobabilistic, stratified purposeful sample [43] of Portuguese mental health professionals—psychologists and psychiatrists—was planned to capture variation and ensure the inclusion of participants in different age groups and work contexts; with different academic degrees and theoretical orientations; and reporting different levels of work experience, knowledge, and use of digital mental health. The sample of participants was identified following referrals from researchers and contacts in the health community. A total of 28 mental health professionals were invited to participate in this study via email or telephone. Of the 28 invited professionals, 15 (54%) replied and agreed to participate. Meaning saturation was established as a stopping criterion, which meant that new participants would not be enrolled once novel fieldwork insights stopped significantly changing the analysis [44]. Data collection ended after 87% (13/15) of participants were interviewed, as meaning saturation was achieved during the last interview.

### Study Context

This study was conducted in the context of the iNNOV Breast Cancer project (ClinicalTrials.gov NCT03275727) [45]. During the iNNOVBC project, we realized that mental health professionals’ attitudes toward digital mental health were significantly associated with previous use of such interventions, potentially affecting program acceptance and implementation. On the basis of this insight, we decided to further investigate the perspectives and practices of mental health professionals regarding the implementation of digital mental health and explore the factors influencing such perspectives and practices using this qualitative interview study.

### Ethical Approval

This study was approved by the ethics review boards of Instituto Português de Oncologia do Porto Francisco Gentil, Unidade Local de Saúde de Matosinhos, Entidade Pública Empresarial, the Portuguese Psychologists Association (Ordem dos Psicólogos Portugueses), and the Portuguese Data Protection Committee (approval number 10727/2017). Written informed consent was obtained from all participants before the onset of study procedures.

### Data Collection

Interviews took place face to face between November and December 2019 at Fraunhofer Portugal AICOS meeting rooms to ensure a private and facilitating environment. A total of 2 interviewers participated in the data collection (CMS and Ana Alves or Elsa Oliveira). Although both interviewers were free to pose questions and provide clarifications, each interviewer was assigned a main role (ie, principal interviewer or observer). The first author acted as the principal interviewer in all interviews. Interviews were conducted in Portuguese.

A pilot interview was conducted at the onset of the study to test the interview guide and train all the researchers on the research protocol. In total, 13 mental health professionals were involved, resulting in approximately 10 hours of audio recordings. The average duration of the interviews was 49 (range 26-81) minutes. All interviews were audio recorded and stored in a pseudoanonymized format in a secure, password-protected location. Interview recordings were transcribed verbatim (CMS and Ana Alves) using oTranscribe (MuckRock Foundation) [46], an open-source transcription web app. Transcripts were coded and analyzed in parallel with data collection to enable data collection to be driven by analysis.

### Researchers’ Characteristics and Reflexivity

The research team comprised outsider, hybrid, and insider researchers with multidisciplinary backgrounds (eg, clinical psychology, engineering, and design). Nevertheless, all data collection team members worked within the field of digital health, which may have influenced the conduction of interviews and analysis. As such, several measures were taken to ensure the validity of the study and promote reflexivity.

Before the start of the study, members of the research team were encouraged to write down their expectations regarding the findings and debate them within the team to elicit preconceptions and prejudiced ideas that could influence the analysis. In addition, data collection co-occurred with data transcription to help reveal areas of the data that required more exploration in subsequent interviews and raise awareness of the potential impact that researchers’ relationship with the research topic and participants could have on data collection and interpretation. During the study, frequent meetings were conducted to encourage reflection and debate on management and interview analysis.

### Analysis

The analysis followed the thematic analysis method of Braun and Clarke [47]. Coding was performed in parallel by 2 researchers (CMS and Ana Alves). After initial familiarization with data through interview transcription and repeated transcript reading, initial codes capturing salient content were independently generated. Scrivener software [48] was used to support the coding of the interview transcripts and the writing of the memos. Regular discussions between the researchers (CMS, Ana Alves, Elsa Oliveira, and FN) were promoted to discuss the results and coding trees, and comparative analyses were performed to ensure reliability. Data patterns were then identified and iteratively organized into themes by clustering and collapsing codes based on similarities and differences. Thematic maps were then assembled and refined as they were applied to the data. Patterns within and across themes were systematically explored and scrutinized until consensus between researchers was achieved, and no additional insights resulted from the analysis of the data. Preliminary and final reports were submitted for participant validation.

## Results

### Participants

A total of 13 mental health professionals participated in the study. Of these 13 mental health professionals, 2 (15%) were psychiatrists, and 11 (85%) were clinical psychologists. Most participants were female (11/13, 85%), and the median age was 35 (IQR 11, minimum 25, maximum 56) years. Participants had a median of 11 (IQR 11) years of professional experience, and most held a master’s degree or higher (9/13, 69%). Most participants were active (12/13, 92%) and working at different universities or research institutions (5/13, 39%), distinct hospitals of the National Health Service (4/13, 30%), and various private practices or charities (3/13, 23%). Most participants (9/13, 69%) developed some sort of clinical practice at the time of the interviews. Approximately 31% (4/13) of participants worked exclusively as clinical and health psychology researchers in the field of digital mental health. Approximately half of our sample had cognitive behavioral therapy orientation (7/13, 54%). Considering the participants’ knowledge and experience using digital mental health, the sample was evenly distributed, including nonusers and occasional and regular users (Table 1). Nevertheless, given the embryonic stage of digital mental health in Portugal [28], half of our sample could be considered as early adopters of digital mental health and thus may not be representative.

**Table 1:** Participants’ sociodemographic characteristics (N=13).

Characteristics			Values, n (%)
**Sex**			
	Female	11 (85)	
	Male	2 (15)	
**Age (years)**			
	23-30	3 (23)	
	31-40	7 (54)	
	41-50	2 (15)	
	51-60	1 (8)	
**Highest academic degree**			
	Licentiate degree	3 (23)	
	Specialization	1 (8)	
	Master’s degree	5 (38)	
	PhD	4 (31)	
**Theoretical orientation**			
	CBT^a^	7 (54)	
	Psychodynamic or existential	2 (15)	
	Humanist	1 (8)	
	Eclectic	2 (15)	
	None	1 (8)	
**Work experience (years)**			
	0-1	1 (8)	
	2-5	2 (15)	
	6-10	2 (15)	
	11-15	5 (38)	
	≥16	3 (23)	
**Work context**			
	Unemployed	1 (8)	
	Private practice or charities	3 (23)	
	National Health Service	4 (31)	
	Universities or research institutions	5 (38)	
**Areas of specialization**			
	Clinical psychology	3 (23)	
	Health psychology	3 (23)	
	Neuropsychology	2 (15)	
	Psycho-oncology	5 (38)	
**Digital mental health self-reported knowledge**			
	Residual	4 (31)	
	Moderate	5 (38)	
	Advanced	4 (31)	
**Digital mental health self-reported use**			
	None	6 (46)	
	Occasional	4 (31)	
	Regular	3 (23)	
**Technology used in clinical practice**			
	Videoconference software (eg, Skype, Zoom, FaceTime, and Doxy)	4 (31)	
	Messaging apps (eg, WhatsApp, Messenger, and Signal)	2 (15)	
	Email (eg, Gmail and ProtonMail)	3 (23)	
	Social networks (eg, Facebook, Instagram, Reddit, and Falar sobre Cancro)	2 (15)	
	Web platforms (eg, Moodbuster, Google Classroom, and Be a Mom)	4 (31)	

^a^CBT: cognitive behavioral therapy.

### Findings

Digital mental health is not addressed at Portuguese universities, being poorly disseminated in health care institutions and frequently limited to private practice and research organizations. As a result, the participants’ understanding of and experience in the field were dissimilar, ranging from sparse to significant and focalized to comprehensive. Although professionals working in private practice were mostly familiar with videoconference counseling and psychotherapy, interviewed researchers had a broader perspective on the various available intervention formats and experience delivering internet interventions and on the web and virtual reality rehabilitation programs. Despite these knowledge and experience decalages, 5 main themes and 16 subthemes became salient in the analysis and characterized the perspectives and practices undertaken by professionals regarding the implementation of digital mental health. These perspectives and practices are listed in the adjacent textbox (Textbox 1) and further described in the following sections.

As the perspectives and practices of participants regarding digital mental health were highly influenced by the purpose of the intervention (psychological assessment, psychotherapy, etc), the selected treatment modality (eg, videoconferencing counseling, internet interventions, etc), and drivers and barriers encountered during the implementation process, the abovementioned practices tended to unfold subsequently but could also co-occur, overlap, be omitted, or assume a recursive nature.

**Themes and subthemes describing participants’ salient perspectives and practices.**

**Themes and subthemes**

**Indication evaluation**

- Assessment of digital mental health uptake drivers
- Appraisal of digital mental health uptake barriers
- Casuistic assessment of the applications and indications of digital mental health

**Therapeutic contract negotiation**

- Clinicians’ credentials presentation
- Therapeutic setting definition
- Therapeutic boundaries framing
- Contingency plan construction
- Privacy, confidentiality, and data protection procedures disclosure
- Legal, jurisdictional, and billing considerations discussion

**Digital psychological assessment**

- Dealing with the absence of validated technology
- Perceived lack of control over the psychological assessment process

**Technology setup and management**

- Adapting and conciliating nonclinical software to clinical purposes
- Developing digital mental health technology

**Intervention delivery and follow-up**

- Appraising the impact of the absence of evidence-based, usable interventions in practice
- Therapeutic alliance establishment
- Perceived lack of control over the therapeutic process

### Indication Evaluation

After hearing about digital mental health from a peer, a conference presentation, or a client requesting to be followed remotely, most participants reacted to digital mental health with disquietude. Having received no formal training on digital mental health, participants presented ambivalent predispositions toward this practice, including “apprehension” (P12) and “curiosity” (P1), and felt the need to further explore whether digital mental health could be applied in their practice:

I felt that there was a grey area in online psychological interventions because there were many questions that were not being answered [...] my idea was I will not go through with this until it is as transparent as possible

P5

Similar to P5, multiple interviewed professionals felt that there was a lack of high-quality guidelines for implementing digital mental health in practice. Professionals had to search thoroughly for legislations and regulations, technical requirements, ethics, and risks of these interventions, and there was an absence of structured intervention frameworks that could be easily adopted. This void required our participants to independently assess who digital mental health could be appropriate for and autonomously delimit digital mental health applications and indications before initiating their digital mental health practice.

According to the participants, digital mental health could be used for prevention, screening, intervention, and rehabilitation purposes. The ubiquitous nature of mobile devices was recognized as an appropriate venue for performing screening and ecological momentary assessments. Complementarily, the low-threshold accessibility, high scalability, and customizable and persuasive design (ie, technology designed for changing users’ attitudes or behavior [49]) of digital mental health could potentially leverage prevention (self-care) interventions and rehabilitation programs. Digital mental health was also considered valuable if integrated within a stepped-care health care model, playing an important preventive and supportive role before escalating to more differentiated health care alternatives:

[Digital Mental Health] makes sense to me [...] precisely before referral to us [psychiatrists] and not exactly for patients with major disorders. That is, it can and should probably be part of healthcare in [...] early stages of illness [...] I don’t want to be too optimistic, but [perhaps] it can effectively prevent future psychiatric illness

P4

During the interviews, it became clear that digital mental health was not for everyone; however, the participants believed that many clients could benefit from this approach. Given the accessibility and convenience of digital mental health approaches, implementation was consensual among geographically isolated clients, migrants, and clients at risk of contracting infectious diseases, such as immunodepressed patients. Digital mental health was also considered to support clients presenting mild to moderate psychological disorders or stabilized major disorders and patients with chronic illnesses, as it could support self-care. Conversely, digital mental health was considered generally contraindicated to clients experiencing severe conditions, such as advanced dementia, psychotic outbreaks, borderline personality disorder, suicidal ideation, or history of parasuicidal attempts, because of a high risk of dropout and the difficulties in managing crisis episodes at a distance. According to the participants, special consideration should also be given to clients living in unsafe environments or experiencing domestic violence, as such conditions could not fulfill the minimum setting requirements to establish rapport, compromising the intervention’s efficacy and making contingency plans hard to deploy remotely. To be able to benefit from digital mental health approaches, participants considered that potential clients should also present minimal literacy and computer skills, have preserved cognitive function, and be motivated and insightful.

In addition to the abovementioned recommendations, interviewees emphasized that digital mental health implementation should be casuistic, and uptake should depend on the assessment of individual, technological, and contextual factors. In certain situations, digital mental health could configure the unique available alternative to provide first aid psychological support, becoming the indicated approach to even manage situations to which it could be typically contraindicated (ie, suicidal ideation):

It’s not that I like it very much, but I have several patients [...] who have emigrated [...] and what happens is that it can be crucial and even vital for that person to know that on the other side of the world there is someone who speaks her language, who understands, and who can [...] help her acquire tools to deal with the situation...

P11

Awareness of such potential led various health care professionals to consider adoption even when their attitudes regarding such modalities were somewhat negative. According to the participants, complying with their perceived duty to provide psychological support to clients in need was of high importance and often superimposed therapists’ preferences, concerns, or digital mental health’s identified limitations, being the triggering point for various participants to start delivering digital mental health interventions.

Another important aspect emerging from the interviews was the notion that digital mental health indications could be dynamic and change during the therapeutic process:

For example, avoidant people, people with social anxiety [...] we may be reinforcing this way of functioning [using an online medium]. Of course, that if we start like this at an early stage and then afterwards, we bring the person in...but if it’s always online and one of the issues is that she’s not able to be face-to-face with other people, we are only reinforcing this behaviour with the online intervention

P13

As explained by P13, digital mental health interventions could be particularly useful in facilitating access and motivating clients for treatment at some point in the process but could also reinforce dysfunction at a later stage, becoming counterproductive. According to participants, the use of digital mental health implied not only assessing the indication of such an approach at the onset of the intervention but also monitoring the impact that the digital model could have on the therapeutic process.

### Therapeutic Contract Negotiation

According to the participants, the delivery of digital mental health interventions should not be a simple transposition of the face-to-face model to the digital format. Appropriate implementations of digital mental health should comply with specific procedural and relational rules that should be clarified and negotiated with clients’ ad initium. Although some participants opted to verbally debate with clients on these rules and procedures, others considered this information to be transposed to a written therapeutic contract formalizing the therapeutic relationship being established:

You were just asking if there were any rules that were established...I believe that in this type of intervention that’s something that should be more demarcated [...] because there is no face-to-face contact. So maybe the intervention should be better delimited, it should be even written, the way things should work [...] the commitment should be established in a more pronounced way.

P6

As noted by P6, the high flexibility and informal character of digital interactions could sometimes collide with the structured setting that clinical interventions require, making mental health professionals uneasy. Consequently, participants felt the need to debate with clients about the nature and format of the proposed intervention.

According to the participants, clinicians’ credentials should be made available to clients, and the digital setting should be clearly defined. Session or module frequency, structure, and length should be communicated to clients at the onset of the intervention, and the importance of client assiduity and compromise should be emphasized to foster client adherence to the digital therapeutic process. The session’s physical space was also a matter of concern. Participants mentioned the importance of guaranteeing a stable and innocuous background environment to underline the professional character of the service being provided. Most participants paid attention to the room dynamic and environmental circumstances in which the session occurred to ensure that no distractions hindered its flow and that confidentiality requirements were met.

Negotiating the therapeutic contract went beyond establishing the setting rules configuring a sort of user manual for both clients and professionals. According to the participants, when using videoconference, specific guidelines should be followed and provided to clients. Defining who was responsible for establishing the connection was considered important to provide clients with assurance. Complementarily, having a waiting room, as provided in some videoconference software, was perceived as important to welcome clients and mimic the face-to-face model.

Professionals also provided orientations regarding lighting and camera positioning to ensure an adequate assessment of clients’ behavior and provided instructions on how to prevent interruptions caused by technology failure. These strategies included turning off other software running in the background and using a cabled network for connection stability. They discussed the diligence to perform in the event of poor audio or image performance, technology failure, or disconnection to avoid impacts on ongoing introspection, ensure adequate therapist feedback, or enable crisis containment. These procedures were perceived as particularly relevant when dealing with crisis situations and became part of a predefined contingency plan. Furthermore, alternative follow-up or communication strategies were identified at an early stage to prevent misunderstandings, promote trust, and make the therapeutic boundaries clear.

Clarifying relationship boundaries during this negotiation phase was capital to most participants:

There may be a tendency in some people to think that because it’s online...[It] is not really a psychologist-client relationship. The dynamics of power may become slightly blurred. So, to mitigate this problem [...] this initial contract dotted the i’s and crossed the t’s [...]. Dual relationships...So...How will we treat each other on social networks, you know? “I will not follow you...I advise you not to do it as well to protect your privacy”...“I will not google you...” that sort of...

P5

To avoid negatively affecting the therapeutic process, most professionals established strict protocols and boundaries. To avoid dual relationships—situations where multiple relational roles exist between a therapist and a client—most professionals exclusively used institutional accounts or platforms to contact clients and refrained from becoming friends or followers of their clients on social media, thus preserving their clients’ privacy and the evolving working alliance.

Professionals also made full disclosures of their data collection and protection procedures, including the storage of personal contacts, health and psychological data, correspondence, or billing data, to assure clients that their privacy was safeguarded. Detailing the costs of each type of interaction (eg, SMS text message, email, and reports) was mentioned as important by participants as it also served the purpose of reinforcing therapeutic boundaries. Finally, our participants discussed the legal and jurisdictional framework that applied to the intervention, especially with clients from abroad and frequent travelers.

### Digital Psychological Assessment

Once the therapeutic relationship was framed, therapists were faced with the challenge of case formulation and adaptation of the psychological assessment process to the digital format. In general, interviewees considered the administration of psychological instruments (standardized questionnaires, projective tests, etc) and clinical interviews digitally approachable. Nevertheless, they had reservations about the potential of remote observation and the possibility of performing an accurate and comprehensive psychological assessment using digital mediums.

Regarding testing, most participants expressed high acceptability of systems capable of supporting remote assessment processes and capable of automatically sending, administering, and scoring tests, underlining their time-saving potential. However, none of the participants used such technology in their clinical practice because of the scarcity of dedicated platforms in the market, limited set of instruments, lack of adaptation to Portuguese and digital contexts, or high subscription costs. Having to overcome such barriers significantly affected professionals’ practices and possibly the psychological assessment process. While some professionals felt compelled to narrow the scope of the evaluation, relying solely on structured clinical interviews to avoid experiencing a high technological burden in their practice or submitting clients to such a burden, others opted to devise alternative ways of administering psychological instruments remotely:

Zoom allowed me to share the screen, so I used to share PQ [a clinical interview] and then clients would see me on the left and the questionnaire on the right and I would fill it out.

P5

Professionals commonly used a collaborative approach to administer structured interviews or questionnaires, using videoconference or emailing encrypted questionnaires to overcome distance and software limitations. However, this approach was not considered adequate for all tests (eg, some neuropsychological instruments), and several doubts were expressed regarding the validity of remotely administering paper-and-pencil instruments. In the absence of instruments duly adapted to the digital context, the administration of psychological instruments raised a feasibility dilemma. Participants were often confronted with the conflicting alternatives of trying to administer the assessment protocol they would implement in person versus implementing the most feasible evaluation protocol while considering usability, time, and investment constraints. Moreover, the security and compliance of such procedures with the General Data Protection Regulation and intellectual property rights were questioned, revealing that some participants were uncomfortable with the strategies they implemented.

Regarding observation, some participants expressed concern over the possibility of losing their “clinical sense” (P4) while performing a remote psychological assessment. Owing to limited vision angles and difficulties in assessing nonverbal communication cues, several interviewees rejected this alternative. Other participants recognized that, although this might be a limitation under some circumstances, in other situations, the digital environment, especially videoconference, could bring an ethnographic dimension to the experience, allowing the therapist to perform a more accurate in loco evaluation of clients’ behaviors and contexts:

Deep down I completely entered her world, which was a room in a house, in an isolated town. I could see the tidiness and untidiness of her little room [...] Often I could see that she was wearing pyjamas [...] and that was a session mobilizer at times because she was in fact very comfortable, but she was also presenting with depressive symptoms [...] And I was there to watch it like a movie. [...] Well, I believe that we might miss some information, but I also think that there is other information, as in this case, that emerges that perhaps in another face-to-face situation would not appear, right? [...] It ended up being somehow invasive of her privacy...(But) That’s right, she was showing it to me.

P6

Digital in loco observation, as described by this participant, provided therapists with the opportunity to gather contextual information that is usually unattainable during in-person appointments, such as the hygiene conditions of their clients’ homes. It also encouraged clients to behave more naturally and, therefore, facilitated the display of clinically significant signs that otherwise could be hidden. However, as clients’ background scenarios and interactions were not under the therapists’ control, accessing contextual information passively provided by clients was sometimes felt by professionals as a violation of their clients’ privacy. Furthermore, the perception of traveling in an unchartered territory could arise, challenging the therapists’ confidence in their web-based assessment capabilities:

I get the impression that...I never know the conditions of the environment where the patient is. I don’t know if someone is listening to the patient or not, I don’t know if what the patient is saying to me is trustworthy or not.

P11

As discussed by P11, the impossibility of guaranteeing communication security and discriminating between all environmental factors potentially affecting clients’ behaviors during digital appointments sometimes threatened therapists’ confidence in using digital mediums for assessment purposes. In addition, the difficulty in discerning if the information conveyed was real or fabricated hindered professionals’ perception of control over the digital psychological assessment process. Overcoming such perceptions required adopting a structured assessment framework capable of anchoring professionals’ practices and supporting them in providing a comprehensive evaluation of the client.

According to some participants, technology could be helpful in providing such a framework and reinforcing professionals’ perceptions of control over this process:

It’s preferable to have a platform...I would rather be framed by a platform, without a doubt...I prefer to shield myself in a situation like this, than not being safeguarded by a platform. I find it more organized, I find it more coherent, I think it makes a lot of sense to have this type of resource.

P6

As mentioned by P6, digital platforms have the potential to integrate several components inherent to the psychological assessment process, facilitate data collection and interpretation, and enable a more comprehensive assessment of the client. Moreover, if designed to comply with data privacy and security requirements, technology could attenuate professionals’ concerns about confidentiality and data breaches, increasing therapists’ perception of control and security while assessing remotely.

### Technology Setup and Management

Being intrinsic to digital mental health, technology plays a determinant role in both psychological assessments and interventions. As each technology (web platforms, mobile apps, chatbots, etc) has its own affordances and characteristics, potentially capturing information and delivering interventions in a distinct way, participants considered that technology should be selected to comply with the characteristics and specific needs of the target population or client. Consequently, professionals were faced with the task of acquiring, adapting, or developing digital technology at some point in the intervention process. The point at which this aspect was addressed depended on the professionals’ work context, training, selected approach, and proficiency in the delivery of digital mental health interventions. Although professionals working with more structured approaches, such as internet interventions, usually addressed this requirement at the onset of the intervention process, therapists working in private practice tended to test and adapt various technologies after a preliminary assessment of the client, making adjustments along the intervention process. Regardless of the adopted strategy (ie, purchasing, developing, or adapting technology), pursuing such tasks was often considered a challenge by professionals.

Confronted with the absence of dedicated technology and the obligation to comply with confidentiality, data privacy, and security requirements, professionals were often forced to conciliate and adapt multiple nonspecific software for clinical purposes:

I developed, within my limitations because I am not a programmer, an encrypted Excel program...I tried my best to develop something [compliant with GDPR] [...] then I bought a cloud [...] which allowed encryption and stored [the data] there. But I struggled a bit [...] Because there’s the excel file and you can put there, some data, but what about the reports? [...] I was forced to handle many different files, all encrypted, with different passwords, then...I had to use a password manager too...And then there’s the e-mail part...I subscribed a platform that allows you to encrypt messages without the recipient using [it] as well. But before, we had to share a password. So, in the first session...I asked people to provide a password for the e-mail communications [...] verbally. Okay, so, I was struggling with that and what I would like to have is a platform where I didn’t need to manage many passwords, where everything is integrated, where I can communicate with the person in a safe way.

P5

Similar to P5, various other participants used nonclinical videoconference software such as Zoom videoconferencing and Skype to conduct remote sessions and administer questionnaires or perform clinical interviews. Encrypted email services were occasionally subscribed to protect therapist-client off-session communication exchanges, and Microsoft Word was used to produce reports. Microsoft Excel was sometimes used to create forms for collecting client data, and some participants mentioned the use of encrypted cloud services for data storage. Implementing and maintaining these technological setups posed a burden on both professionals and clients and compromised therapists’ efficiency and satisfaction. Thus, most interviewees agreed on the importance of developing dedicated and comprehensive platforms to implement digital mental health interventions efficiently and cost-effectively.

However, many participants considered that developing digital mental health technology was a “long and challenging process” (P10). According to some participants, there is a tendency to design “one size fits all” (P13) tools and interventions; however, digital mental health technology should capture and portray emotional and relational nuances to fit different clients. Trust and transparency should be embedded in the structure of the software, and empathy should be conveyed through developed assessment and intervention materials. Digital mental health technology should be usable, inclusive, customizable, scalable, and disseminated, but also culturally sensitive, and assume a personalized and dynamic form. Complying with these requirements was considered highly demanding, especially when developing intervention programs such as internet interventions and web-based or virtual reality rehabilitation programs.

According to some interviewees, developing assessment and intervention programs requires the assembly of a multidisciplinary team. Although mental health professionals need to create the content, designers could be required to work on the graphical presentation, and developers could be needed to create a vehicle to deliver the intervention. Our participants also stated that developing digital mental health programs usually entailed performing comprehensive literature reviews and user research (eg, based on interviews, observations, and usability testing) to identify the best medium for treatment delivery and understanding how to structure the content and exercises to be included in the program under development. As a rule of thumb, participants stated that digital mental health programs should be adaptable and responsive to different formats and devices to reach most clients in ways they find appropriate. However, fulfilling this criterion is not always possible mainly because of financial and technical limitations:

In an ideal context we could have developed a web and a mobile app version that complemented each other, but at the time it was not possible. So, we had to decide, and the web version seemed more viable to us, it was...[cheaper] and it allowed us to have the content the way we wanted. An app required...shorter content, a different organization that for us was more difficult to develop in a first rehearsal of the program.

P8

Creating intervention content was also considered a challenging process. As identified by P8, it required understanding how the characteristics of a selected technology could affect the therapeutic program being developed. The text length, type of audiovisual content, exercises, and features that can be included in each program vary depending on the selected technology, and the impact that its limitations could have on treatment efficacy should be considered. Furthermore, developing interventions’ content implied writing to hypothetical personas [50] and steering development to fit clients’ characteristics. However, as the act of typifying clients often collided with the casuistic approach most professionals were trained in and adopted in clinical practice, various participants mentioned feeling uncomfortable with such an approach:

I found this adaptation very difficult because we know that the strategies are suitable for each person and what works for one, may not work with another. “But how do we get this into the material?” So, what I tried to emphasize was “these are just suggestions, the most important thing is to follow what makes you feel more comfortable, and you should adapt it according to what makes sense to you,” and I underline “don’t look at this as laws and rules.” Specially because when suggested strategies don’t work and everything falls apart, patients who follow all the steps may feel cheated, or question their self-efficacy, their skills...[...] So, my biggest concern was not to cause more damage than people were already experiencing.

P13

As discussed by P13, developing digital mental health interventions and tools was considered a major responsibility. This implied not only designing personalized evidence-based empathetic programs but also acknowledging the impact such programs could have on clients beforehand. The different possible intervention outcomes had to be anticipated, and strategies to ensure that possible adverse effects were prevented, monitored, and addressed should be integrated into developed interventions to comply with safety and beneficence requirements. In this regard, iterative testing was considered crucial not only to identify unanticipated characteristics of the program with the potential to negatively affect clients but also to guarantee programs under development were “culturally sensitive and adapted to the target-population” (P9).

To achieve such a high level of technology refinement, participants referred to the different development stakeholders—academia, industry, end users, and funding bodies—that needed to be aligned, and that the multidisciplinary development team should be able to communicate effectively, adapt, and collaborate. However, communication within development teams could be difficult because of different backgrounds within the team, dysrhythmic development processes, and the existence of divergent goal-steering development. This “misalignment” (P7) could significantly affect the quality, usability, and sustainability of developed interventions. As a result, an important gap in what concerns high-quality, evidence-based digital mental health technology was identified by participants.

### Intervention Delivery and Follow-up

During the interviews, the “absence of evidence-based ready to use interventions” (P4) and the lack of accessible, secure, and comprehensive assessment and treatment tools were identified as the major factors affecting treatment delivery. According to the participants, if not properly designed, handled, and monitored, technology could be experienced as a “barrier” (P7) or “filter” (P6), especially by clients with limited sensory, cognitive, and physical user capabilities. Various participants recognized the lack of usability of existing tools and programs as an important implementation obstacle. Interventions’ “high complexity both in terms of structure and content” (P10), as well as the use of noninclusive design approaches, were considered problematic because of the deleterious impact they could have on treatment adherence, outcome, and rapport.

As perceived by various interviewees, establishing a therapeutic alliance on the web is feasible, and the quality of the established bond may be equivalent to that occurring in face-to-face interactions. However, participants considered that such a process could be affected by poor technology design, adaptation, or failure, and specific strategies should be implemented to facilitate rapport.

I did not feel much difference, to be honest, between presential and online interventions in terms of therapeutic alliance [...] you can create the bond in the same way. [...] in the first sessions, I keep assessing if and how the person feels me present, so to speak...If we feel connected and sometimes when I realize that the person is looking at the camera a lot, I give the eye contact suggestion “can you distance yourself from the screen, place the program window in a different way so that we can both be looking at each other in the eye without having to be looking at the camera.” [...] It works, and I feel well, I feel in tune with the person, something that I thought I would feel more in person.

P5

According to the participants, fostering the therapeutic alliance on the web required close monitoring of both the relational and technological dimensions of the therapeutic process. As mentioned by P5, technical instructions such as adequately distancing and positioning the camera or repositioning tabs and windows on the computer desktop while videoconferencing should be followed to ease eye contact, bring authenticity to digital interactions, and strengthen the working alliance being established. In other intervention formats, such as internet interventions, our participants emphasized the importance of using different communication channels (eg, written, verbal, and visual) to convey warmth and empathy during the intervention and provide timely and personalized feedback to the client regarding homework assignments or intervention strategies being implemented. According to participants, the interventions’ materials and therapists’ written feedback should be made permanently available to extend the therapeutic setting beyond booked appointments, reinforce the evolving working alliance, and potentially accelerate behavior change. The conduction of close follow-up sessions was also identified as important to “promote relationship continuity” (P1). Various participants suggested that an initial face-to-face appointment was important as well to “give a push to the bond and compromise being established” (P3). Nevertheless, the development of such connection could be influenced by clients’ personal characteristics—namely, age, information and communications technology literacy, and cultural aspects. Therefore, close monitoring of the evolution of the working alliance should be performed, and, if necessary, alternative and simpler communication mediums, such as face-to-face appointments or telephone calls, should be used to prevent treatment abandonment.

Client dropout or early abandonment was a recurrent concern captured in the interviews, particularly in the context of crisis situations. Digital mental health was regarded as an unchartered territory in what concerned managing crises, as most participants stated not being prepared to handle such situations remotely and required specific training on how to build contingency plans and how to detect risk situations early:

Whoever is on the other side must be aware, know [the signs], and be able to detect the moment when risk arises, right? And despite being at a distance [...] even when in different countries, [the therapist] must be able to properly assess and refer the client. But I don’t know how much we can contain on this side...

P3

Some of the strategies implemented by participants in this regard included restricting the use of digital mental health to mild or moderate conditions, collecting emergency contacts at the onset of the intervention, and mapping local institutions to be activated under crisis circumstances. Nevertheless, because of the limited action range therapists felt in these situations, various participants were hesitant to work exclusively online, and most refused to consult with anonymous clients.

Delivering digital mental health interventions implied “dealing with unforeseen challenges along the therapeutic process” (P2) without having clear guidelines and training on how to pursue such practice and how to deal with problems such as managing adverse events or crises at a distance. Therefore, a perception of a lack of control over the therapeutic process was experienced by some therapists. This perception was reinforced by the generalized notion that information and communications technology systems are susceptible to security and confidentiality breaches and discouraged therapists’ sustained adoption of digital mental health.

Perception of control seemed to be influenced as well by the intervention format and type and frequency of communication established between therapist and client. Ranging from residual in unguided interventions to augmented in blended care interventions, therapists’ perception of control seemed to increase when guidance was provided, and synchronous interactions with clients occurred:

In unguided interventions, our concern relates to the fact that we don’t have control over the evolution of the symptoms and the fact that a person can give up anytime...although, this can happen in presential sessions as well, right? But online we have less information about what’s happening on the other side and this can somehow be a matter of greater concern...I don’t think it’s an impediment, but it is perhaps the issue that concerns me the most [...] being more frequent [...] half face-to-face sessions and half online sessions, it would allow greater progress monitoring... a greater sense of control in some way, even though this perception might be subjective...

P8

According to P8, unguided formats of treatment delivery or guided interventions, including exclusively asynchronous communications with clients, could make a proper case formulation difficult and compromise the assessment of treatment outcomes, hindering therapists’ perception of control over the therapeutic process and making them refuse or hesitant to use such formats. Consequently, most participants endorsed blended care interventions.

To be encouraged to implement digital mental health interventions, particularly formats other than blended care, various participants referred to the need for structured training on digital mental health:

We must have the necessary knowledge and practice to be comfortable working online [...] otherwise my concern will be “I can’t do this, I can’t do that” and my attention is no longer on the person, on the questionnaire’s results and I believe the usefulness of these tools is lost

P12

Training was considered instrumental in providing therapists with the necessary knowledge and practice to implement digital mental health interventions confidently. However, the absence of formal digital mental health training programs was transversally identified as a major gap affecting its adoption by professionals.

## Discussion

### Principal Findings

This study suggests that mental health professionals deemed important or engaged in the following practices while implementing digital mental health interventions: (1) indication evaluation, (2) therapeutic contract negotiation, (3) digital psychological assessment, (4) technology setup and management, and (5) intervention delivery and follow-up. Although these practices tend to unfold subsequently, they could also co-occur, overlap, be omitted, or assume a recursive nature depending on the purpose of the intervention, the selected treatment modality, and the drivers and barriers encountered along the implementation process.

The implementation of digital mental health started with an evaluation of its indication to a given client and involved the appraisal of individual, technological, and contextual aspects. Similar to previous research, our participants perceived digital mental health as indicated only to a subset of clients [26], restricting it to individuals presenting with mild to moderate symptoms [33,51], limited comorbidity [39], and low risk [41]. However, such recommendations were not consensual among all participants in this study, and in reflection, it may be non–evidence based and unethical [52]. A previous meta-analysis showed that digital mental health interventions could be efficacious for individuals presenting with severe symptoms and suicidal ideation, and there is little evidence that these groups present an increased risk of adverse events [53,54]. Furthermore, a recent publication [55] disclosed that at MindSpot, one of the world’s largest publicly funded web-based clinics, users’ mean symptom scores were in the moderate to severe range, and a quarter presented with suicidal ideation. This reality suggests that digital mental health might be an important mental health care gateway for clients experiencing severe conditions, and as claimed by various participants, a casuistic indication assessment should be performed to comply with equity and beneficence requirements [56].

Professionals’ perceived duty to provide support to clients in need was an important digital mental health adoption driver identified in this study, which has been poorly explored in the literature [32]. Such responsibility often superimposes participants’ preferences, concerns, and digital mental health’s perceived limitations, which is the triggering point for various participants to start delivering digital interventions. Digital mental health’s low-threshold accessibility and convenience [28] reinforced such decision, particularly when treating clients living in geographically underserved areas [51] and patients who were mobility impaired [29], a finding that corroborates previous research [26]. However, due to digital mental health’s indication dynamic character [57], such an approach was generally not perceived as a standalone alternative but as part of a continuum where different types and degrees of interactions between client and therapist could be operated to fulfill the clients’ best interests. As such, digital mental health interventions were frequently understood as potentially following, intersecting with, or culminating in other treatment approaches (eg, face-to-face interventions and pharmacotherapy), better fitting a hybrid mental health care model [58].

However, hybrid mental health care models have been insufficiently addressed in the literature [26], and a lack of structured intervention frameworks presenting clear guidelines on how to implement digital mental health in an ethical, legal, and secure way was transversally identified by participants as a barrier compromising its implementation. Similar findings have been reported in previous studies [33-36]. To compensate for this lack of structure, professionals independently formulated rules and procedures to organize their digital practice, placing great emphasis on the negotiation of the therapeutic contract.

This practice often involved discussing with clients, in a highly structured manner, digital setting rules, confidentiality and data protection procedures, therapeutic boundaries, and contingency plans to be deployed in potential crisis situations. Despite generalized agreement on the importance of debating the abovementioned aspects with clients, various participants were concerned about the impact such high formality could have on the therapeutic process. To the best of our knowledge, this is the first study to document the digital therapeutic contract negotiation procedure; therefore, further research should be performed to assess its impact.

After framing the therapeutic relationship, professionals typically proceeded with digital psychological assessment. Although the methods they used were similar to the in-person process (eg, observation, testing, and clinical interviews), participants reported that this process was highly dependent on technology availability, characteristics, and performance. During the digital psychological assessment, difficulties in assessing nonverbal communication cues became salient, and the inability to fully control the evaluation setting raised concerns over the possibility of performing an accurate and comprehensive psychological assessment on the web. Such concerns echo the findings of previous research [28,35,59,60]. In a recent study, Mendes-Santos et al [28] reported that approximately 60% of psychologists perceived remote psychological assessment processes as inaccurate, increasing the possibility of misdiagnosis. In another study by Gilmore and Ward-Ciesielski [60], 30% of participants identified digital assessment as risky, particularly when evaluating clients at a high risk of suicide. Nevertheless, previous studies have validated telephone-based behavioral assessments [61], and the equivalence between paper-and-pencil and web-based testing has been consistently documented [62,63]. More importantly, a previous study by Godleski et al [64] reported that suicide risk assessment can be effectively completed via videoconference and in-home messaging devices and is as effective as suicide risk assessment completed in person. This discrepancy between participants’ stance toward digital mental health and evidence has been previously identified in other studies [28,34] and suggests that an important knowledge gap in this domain hinders professionals’ adoption of such an approach.

Another important obstacle identified in this study, which potentially compromised professionals’ performance and, therefore, their sustained adoption of digital mental health [40], was the absence of usable, validated, and affordable digital mental health technology. Although these factors have been singled out as potential barriers compromising digital mental health adoption in previous research [65], their real impact on clinical practice is undetermined. Moreover, the strategies used by professionals to overcome such obstacles have been poorly explored. This aspect may be justified by a greater number of studies focusing on specific research trials [22,29,39,41] or web-based clinics that evolved from research programs [38]. As the technological infrastructure required to deliver interventions in these contexts is assembled beforehand, professionals are possibly spared from the difficulties of acquiring, adapting, and developing digital mental health technology.

Nevertheless, the implementation of digital mental health necessarily entails such procedures. Interestingly, the point at which this technology setup occurred depended on the professionals’ work context (eg, research and clinical settings), training, selected treatment approach, and proficiency in the delivery of digital mental health interventions. Professionals developing regular digital practices or working with more structured approaches usually addressed this requirement at the onset of the intervention process, consistently using the preselected technological setup along the process. Conversely, therapists making sporadic use of digital mental health tended to blend in-person and digital approaches more often, frequently electing preferential technology for treatment delivery after a preliminary assessment of the client. The tendency to test and adapt various technologies during the intervention process also characterized the latter group. This practice is possibly justified by a confrontation with technology limitations during the intervention, which were initially unforeseen, or the dynamics of the treatment process requiring different technology affordances to be explored according to clients’ progress.

Challenged with the limited availability of affordable evidence-based tools capable of comprehensively supporting treatment delivery, professionals were often forced to narrow the scope of their clinical work or devise alternative ways of pursuing such practice. Mirroring other professionals’ procedures [66,67], the adaptation of nonclinical software (eg, Gmail and Zoom videoconferencing) for psychological evaluation and intervention purposes was a strategy frequently adopted by participants in this study. As such practice risked noncompliance with the General Data Protection Regulation, intellectual property rights, and good clinical practices, various participants were hesitant to make sustained use of digital mental health, reserving it for extreme situations such as providing support to migrants. In addition, most professionals underlined the importance of expediting the development of evidence-based digital mental health technology capable of framing therapists’ digital practices. This potential of technology to structure the clinical process was also found in previous studies [40,68].

Nonetheless, the development of digital mental health technology was often perceived as long, complex, and expensive. Moreover, a misalignment between development stakeholders (eg, terminology, development priorities, and quality assurance criteria) was identified as a major barrier compromising the effectiveness, usability, and transference of developed programs into clinical practice. Such perceptions align with the current discussion around digital mental health development and implementation processes. Previous publications have discussed the need to turn such processes more agile [69], solution focused [1], and integrated into clinical practice [8,70]. Moreover, a paradigm shift toward the design of digital mental health services instead of products seems to be unfolding [1,55]. According to Mohr et al [1], to be widely adopted and fully integrated into health care systems, digital mental health services need to be designed to fit into the fabric of clients’ lives, respect professionals’ workflows, and be able to accommodate changes in the care environment and technological ecosystem. If not, digital mental health might be experienced as an added burden rather than as an added value, as became salient in this research. Similar results have been reported by Cerga-Pashoja et al [40].

During intervention delivery and follow-up, professionals were often concerned about the negative impact of nonusable, validated, or defective technology on treatment adherence, outcome, and rapport. Similar to other studies, various participants considered that a positive therapeutic relationship could be established and extended on the web [29,57], potentially accelerating the treatment progress [32]. To be able to foster and monitor the therapeutic alliance, most participants recognized that the development of new relational and technical skills (eg, the management and development of digital technology and engaging with clients remotely) was necessary, a finding that corroborates previous research [39,55].

However, professionals’ lack of training in digital mental health has been transversally identified in this and previous studies [28,34,66], and most participants relied solely on research and trial and error experiences of use to develop such skills. As a result, most professionals questioned their self-efficacy [39] to assess and intervene remotely, and a marked perception of lack of control over the implementation of digital mental health interventions prevented them from fully adopting such an approach. This perception of lack of control has been documented before in relation to technology [39], therapeutic settings [71], therapeutic processes [56], and management of crisis situations [35]. However, no study deeply explored this barrier. Adding to previous research, insights from this study suggest that perceptions of lack of control over case formulation; the assessment of treatment outcomes; and the detection, management, and monitoring of treatment adverse events also discouraged adoption. Moreover, professionals’ perception of control seemed to be highly influenced by their experience of use and training. As such, capacitating therapists with the necessary skills to implement digital mental health interventions proficiently seems key to building confidence within the class and expediting adoption.

Unfortunately, there is currently no standard method of training therapists in digital mental health, and structured training and supervision initiatives are limited [72]. Moreover, the paucity of research assessing the impact of such programs is concerning [73]. Considering that the skills and knowledge required for effectively delivering digital mental health interventions are considerably different from those required in traditional models of care [55], not addressing this gap menaces both the quality of interventions delivered by untrained professionals, as well as the future sustained adoption of digital mental health.

### Strengths and Limitations

This study had various strengths. It involved a purposeful selection of participants with different sociodemographic characteristics, working within different contexts, presenting different levels of knowledge and use of digital mental health, providing an in-depth understanding of the perspectives and practices of mental health professionals regarding digital mental health. Furthermore, data collection was theory informed and co-occurred with data transcription, helping to reveal areas of the data that required more exploration in subsequent interviews, enriching the data set. Continuous reflection and a consensus approach were also adopted during data collection and analysis. This practice raised awareness of the potential research biases affecting the study. Finally, respondent validation and investigator triangulation were used to increase the validity of the results.

Nevertheless, a few study limitations must be considered when interpreting our findings. Although a stratified purposeful sample was selected to capture variation, as in many interview studies, the representativeness of the sample cannot be established. The results are based on an in-depth analysis of interviews provided by a small sample of Portuguese mental health professionals and, therefore, may not be transferable to other contexts. The fact that digital mental health is still at an embryonic stage in Portugal [25] might have influenced participants’ attitudes and opportunities to explore digital mental health—namely in what concerns other digital mental health modalities and technologies (eg, artificial intelligence, serious games, wearable devices); therefore, it is unclear whether the same issues would be identified among professionals practicing, for example, in digital mental health frontrunner countries [74]. Another aspect to consider is that the main interviewer was previously known to most participants from her role as a digital mental health researcher or clinical psychologist. This fact may have introduced a social desirability bias, possibly leading interviewees to be less critical of digital mental health. Finally, this study failed to pursue other forms of triangulation, such as method, theory, and data source triangulation, which would have been advisable to test the validity of the obtained results and gain a more comprehensive understanding of the implementation of digital mental health. However, previous research seems to indicate that the insights from this study have ecological validity [28].

### Conclusions and Future Research

This study aimed to characterize in depth the perspectives and practices of mental health professionals regarding the implementation of digital mental health and explore the factors influencing such perspectives and practices. Our findings suggest that mental health professionals deemed important or engaged in the following practices to implement digital mental health interventions: (1) indication evaluation, (2) therapeutic contract negotiation, (3) digital psychological assessment, (4) technology setup and management, and (5) intervention delivery and follow-up. Digital mental health’s low-threshold accessibility and professionals’ perceived duty to provide support to their clients were identified as the main drivers facilitating implementation. Conversely, the lack of structured intervention frameworks; the unavailability of usable, validated, and affordable technology; and the absence of structured training programs negatively affected implementation and, consequently, professionals’ adoption of digital mental health.

To overcome the abovementioned barriers and expedite professionals’ adoption of digital mental health, the publication of legal, regulatory, and practice frameworks that can be easily transferred to practice seems necessary. These guidelines could be conjointly elaborated by different digital mental health ecosystem stakeholders (eg, policy makers, regulatory bodies, clinicians, and information technology and data protection specialists) to ensure that they are comprehensive enough to provide the necessary structure professionals require to confidently work remotely. Moreover, the co-development of digital mental health technologies and services must be encouraged. Clients and professionals must participate in the development process to guarantee that such services answer their most pressing needs and integrate smoothly into routine care. In this context, user and implementation research is key to streamlining the development and implementation processes. Finally, to guarantee proper implementation of digital mental health, the design of training programs that are structured according to professionals’ most pressing needs and that are assessed, certified, and made widely available to professionals seems of high importance. To incentivize adoption, professionals must be trained in the implementation of digital mental health interventions and in managing the main barriers affecting it. Research on professionals’ current unmet digital mental health training needs is necessary to structure this process. Addressing these research action axes could not only empower professionals in the delivery of digital mental health interventions but also expedite adoption and help close the current mental health care treatment gap affecting health care systems worldwide.

## Appendix

Multimedia Appendix 1 Mental health professionals’ semistructured interview script.

## Abbreviations

COREQ: Consolidated Criteria for Reporting Qualitative Research
